# Predictive factors for permanent hypoparathyroidism following total thyroidectomy: A retrospective cohort study of 5,671 cases

**DOI:** 10.20945/2359-4292-2024-0379

**Published:** 2025-03-24

**Authors:** Daniela Tamega Joaquim, Hugo Fontan Kohler, José Guilherme Vartanian, Luiz Paulo Kowalski, Genival Barbosa de Carvalho

**Affiliations:** 1 Departamento de Cirurgia de Cabeça e Pescoço e Otorrinolaringologia, A.C.Camargo Cancer Center, São Paulo, SP, Brasil; 2 Departamento de Cirurgia de Cabeça e Pescoço e LIM 28, Faculdade de Medicina da Universidade de São Paulo, São Paulo, SP, Brasil

**Keywords:** Thyroid, thyroidectomy, complications, permanent hypoparathyroidism, risk factors, prevention

## Abstract

**Objective:**

To evaluate the rates of permanent hypoparathyroidism based on demographic variables, patient comorbidities, clinical staging of the disease, surgery performed, and severity of transient hypoparathyroidism.

**Subjects and methods:**

This is a retrospective cohort study with patients who underwent total thyroidectomy with or without neck dissection between January 2014 and December 2021.

**Results:**

5,671 patients were analyzed, 966 (^17^)%) presented transient hypoparathyroidism and 106 (1.8%) developed permanent hypoparathyroidism. The logistic regression model analyzing the cohort of patients with transient hypoparathyroidism demonstrates that the number of dissected lymph nodes from the central compartment, immediate postoperative PTH levels, the necessity of postoperative intravenous (IV) calcium supplementation and the duration of IV calcium supplementation are significant predictors. When applied to the original dataset, this model presents a NPV of 1.0000 and a PPV of 0.9594 with an overall accuracy of 0.9624.

**Conclusion:**

The incidence of permanent hypoparathyroidism was closely associated with the extent of level VI dissection, particularly regarding the number of lymph nodes removed and whether the dissection was bilateral. Furthermore, the severity of post-operative hypocalcemia was demonstrated by the decrease in PTH levels, as well as the need for and duration of intravenous calcium supplementation.

## INTRODUCTION

Hypoparathyroidism is the most common compli- cation following total thyroidectomy and can be classified into two categories: transient, when the need for calcium replacement lasts up to 6 months after sur- gery, and permanent, when this need extends beyond 6 months (^1^). The incidence of hypoparathyroidism var- ies widely in the literature, with transient hypoparathy- roidism reported between 4.7% and 24%, and permanent hypoparathyroidism between 1% and 5.7% (^2,3^).

Trauma to the parathyroid glands and their vascular supply during surgery is the primary cause (^4,5^) and is influenced by the surgeon’s experience, neck dissection performance, and preoperative condition. It may also be linked to factors such as female sex, body mass index (BMI), diabetes mellitus, serum parathyroid hormone (PTH) levels, the number of parathyroid glands identified and removed, thyroiditis, thyroid volume, low preoperative vitamin D levels, and previous bariatric surgery (^4,5,6,7,8,9,10,11,12,13^).

The management of transient hypoparathyroidism primarily involves calcium and calcitriol replacement and is usually overseen by the head and neck surgeon. In contrast, the treatment of permanent hypoparathyroidism is more complex and generally managed by an endocrinologist, due to the risk of severe complications like hypocalcemia crises and neuropsychiatric and gastrointestinal diseases, in addition to the potential loss of renal function from chronic calcium supplementation (^14,15^).

Despite being different aspects of the same condition, the management for transient and permanent hypoparathyroidism differs significantly. While transient hypoparathyroidism has been extensively studied in terms of risk factors, controversies still exist regarding the predictive factors for permanent hypoparathyroidism, which critically affects the long-term quality of life of patients undergoing total thyroidectomy (^16,17,18^).

Thus, analyzing the predictive factors for permanent hypoparathyroidism is invaluable in clinical practice to identify patient subgroups that may benefit from early intervention, thereby minimizing the long-term morbidity associated with the disease. Accordingly, this study aims to evaluate the predictive factors for permanent hypoparathyroidism based on demographic, clinical, and pathological variables in patients undergoing total thyroidectomy at a single tertiary institution.

## SUBJECTS AND METHODS

All patients who underwent surgical procedures were prospectively included in an institutional database. We selected patients who underwent a total thyroidectomy between January 2014 and December 2021. All patients were eligible for inclusion, except those with prior parathyroid disease or calcium metabolism disorders. Follow-up information was last updated in December 2023. The study received approval from the Research Ethics Committee, under CAAE approval number 5.973.982.

A standard form was used for data collection and included information on age, sex, tobacco and alcohol consumption, BMI, comorbidities, pathological diagnosis and stage for malignant disease, presence of extrathyroidal extension, parathyroid gland removal and implantation, and the performance and extent of the neck dissection. The primary outcome was permanent hypoparathyroidism, defined as the persistence of PTH levels below the reference level for more than six months after surgery, necessitating oral calcium and calcitriol supplementation.

The data were processed using SPSS Statistics v. 20.0 (IBM, USA) and R. Continuous variables were summarized by mean and standard deviation, with centering and scaling prior to model inclusion. Mean comparisons were conducted using Student’s t-test, and proportions were compared using the chi-squared test. The number of removed parathyroid glands and T classification were treated as ordered factors, with cT classification set to 0 in patients with benign disease. A logistic regression model was constructed to develop a predictive model for the outcome from independent variables.

Model performance was assessed through diagnostic metrics, such as sensitivity, specificity, negative predictive values (NPV) and positive predictive values (PPV), and the receiver operating curve (ROC). For transitional hypoparathyroidism, pre-surgical variables were considered, while, for permanent hypoparathyroidism, post-surgical variables were also included. Given that only patients with transitory hypoparathyroidism can develop permanent hypoparathyroidism, this subset was specifically analyzed for the transition. Models utilized a bootstrap approach with stepwise variable selection, based on the Akaike information criteria, across 1,000 replications and were validated on the original dataset. A p-value < 0.05 was considered statistically significant.

## RESULTS

After applying the inclusion and exclusion criteria, we included 5,671 consecutive patients in our analysis. Of these, 4,424 (78%) were female, with a median age of 44 years. No comorbidities were present in 4,522 (79.7%) of the patients (Table 1). The preoperative diagnosis of malignancy was established in 4,262 patients (75.2%). Neck dissection was performed on 794 patients (14%): unilateral level VI in 454 patients (8%); bilateral level VI in 78 patients (1.3%); unilateral levels II to VI in 242 patients (4.3%); and bilateral levels II to VI in 18 patients (0.3%) (Table 2).

**Table 1:** Demographic and clinical data for the entire cohort and for patients with transient hypoparathyroidism

variable	Value	Complete cohort	Cohort with transitory hypoparathyroidism
Age	Mean (SD)	45.2 (13.6)	43.6 (12.9)
Gender	Female	4,424 (78.01%)	800 (82.82%)
	Male	1,247 (21.99%)	166 (17.18%)
Payment modality	Public	426 (7.51%)	77 (7.97%)
	Insurance	5,191 (91.53%)	878 (90.89%)
	Private	54 (0.95%)	11 (1.14%)
cT classification	cT0	1,540 (27.15%)	250 (25.87%)
	cT1	3,521 (62.09%)	586 (60.66%)
	cT2	477 (8.41%)	95 (9.83%)
	cT3	121 (2.13%)	32 (3.31%)
	cT4	12 (0.21%)	3 (3.10%)
cN classification	cN0	5,329 (93.97%)	838 (86.75%)
	cN1a	67 (1.18%)	22 (2.28%)
	cN1b	196 (3.46%)	82 (8.49%)
	cNx	79 (1.39%)	24 (2.48%)
Body mass index	Mean (SD)	27.5 (6.0)	27.6 (5.8)

**Table 2:** Demographic and clinical data for patients with transient and permanent hypoparathyroidism

Variable	Value	Cohort with transitory hypoparathyroidism	Cohort with permanent hypoparathyroidism
Age	Mean (SD)	43.6 (12.9)	41.8 (12.6)
Gender	Female	800 (82.82%)	88 (83.02%)
	Male	166 (17.18%)	18 (16.98%)
Payment modality	Public	77 (7.97%)	6 (5.66%)
	Insurance	878 (90.89%)	100 (94.34%)
	Private	11 (1.14%)	0 (0.00%)
cT classification	cT0	250 (25.87%)	22 (20.75%)
	cT1	586 (60.66%)	67 (63.21%)
	cT2	95 (9.83%)	12 (11.32%)
	cT3	32 (3.31%)	4 (3.77%)
	cT4	3 (3.10%)	1 (0.94%)
cN classification	cN0	838 (86.75%)	91 (85.85%)
	cN1a	22 (2.28%)	1 (0.94%)
	cN1b	82 (8.49%)	10 (9.43%)
	cNx	24 (2.48%)	4 (3.77%)
Body mass index	Mean (SD)	27.6 (5.8)	27.0 (5.7)
Number dissected central lymph nodes	Median (range)	0 (0-48)	1 (0-48)
Postoperative PTH	Mean (SD)	9.87 (12.09)	5.23 (6.63)
IV calcium reposition	No	923 (95.54%)	63 (59.43%)
	Yes	43 (4.45%)	43 (40.57%)
Duration of IV calcium reposition		0 (0-12)	0 (0-12)

IV: intravenous

Of the 5,671 patients analyzed, 966 (17.0%) experienced transient hypoparathyroidism, and 106 (1.8%) developed permanent hypoparathyroidism. Of these, 88 (83%) were male, with a median age of 40.5 years. A total of 86 (81.1%) patients had comorbidities, with 6 (5.7%) being smokers, 4 (3.8%) former smokers, and 7 (6.6%) alcohol drinkers. Neck dissection was performed on 33 patients (31.1%), with 11 (10.4%) undergoing unilateral level VI, 13 (12.3%) bilateral level VI, 2 (1.9%) unilateral levels II to VI, and 7 (6.6%) dissections at other levels. The extent of neck dissection was identified as a statistically significant predictor of the risk factor for definite hypoparathyroidism (*p* < 0.001).

Intravenous calcium replacement was required for 43 (40.6%) patients, and 19 (17.9%) needed a continuous infusion pump. The necessity for intravenous calcium replacement statistically significantly predicted the risk of definite hypoparathyroidism (*p* < 0.001).

**Figure 1:**
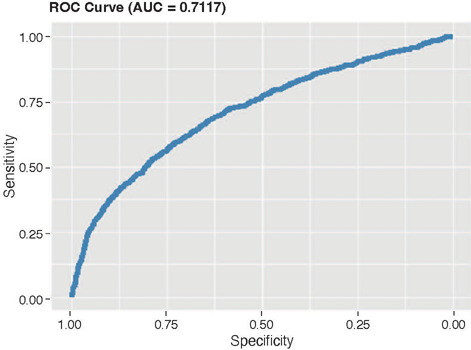
ROC curve for transitory hypoparathyroidism after a logistic regression model using a bootstrap approach and validation in the original dataset.

The logistic regression model for permanent hypoparathyroidism identified sex, age, BMI, cT classification, and preoperative lymph node metastasis location as significant predictors. This model exhibited a NPV of 0.5839 and a PPV of 0.8255, and an overall accuracy of 0.8188 when applied to the original dataset. The ROC curve for this model is shown in Figure 1.

**Figure 2:**
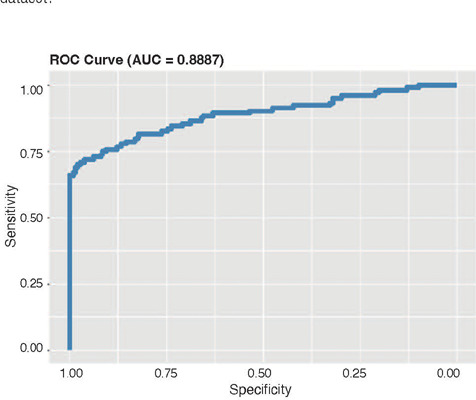
ROC curve for permanent hypoparathyroidism after a logistic regression model using a bootstrap approach and validation in the cohort of patients with transitory hypoparathyroidism.

The logistic regression model analyzing the cohort of patients with transient hypoparathyroidism found the number of dissected lymph nodes from the central compartment, immediate postoperative PTH levels, the necessity for postoperative intravenous (IV) calcium supplementation, and the duration of this supplementation as significant predictors. Applied to the original dataset, this model yielded an NPV of 1.0000, a PPV of 0.9594, and an overall accuracy of 0.9624. The ROC curve for this model is presented in Figure 2.

## DISCUSSION

In the present study, the incidence of permanent hypoparathyroidism was comparable to that observed in other studies (^7,11,12,13^) and was closely associated with the number of lymph nodes dissected from the central compartment, reflecting the extent of the surgical procedure. It was more commonly observed in cases involving bilateral level VI dissection. Other contributing factors included immediate postoperative PTH levels, the requirement for IV calcium supplementation, and the duration of IV calcium therapy. These findings collectively underscore the correlation between the extent of surgical intervention and the increased risk of permanent hypoparathyroidism.

During level VI cervical dissection, preserving the identified parathyroid glands and avoiding ligation of the inferior thyroid artery when possible is recommended to minimize the risk of ischemic injury(^5^). For the inferior parathyroid glands, in cases of uncertainty about their identification in the specimen from level VI dissection, intraoperative frozen section analysis can be utilized when available for confirmation. If their vascular supply cannot be maintained, the glands should be transplanted into the cervical muscles to ensure optimal viability.

Confirming that the extent of surgery is a determining factor in permanent hypoparathyroidism, Unlu and cols. (^11^) conducted an analysis comparing total thyroidectomy to thyroidectomy associated with central neck dissection. They identified higher rates of transient hypoparathyroidism (52.2% *vs.* 20.5%, *p* = 0.000) and permanent hypoparathyroidism (5.8% *vs.* 0.9%, *p* = 0.064), respectively. The relative risk (RR) of central neck dissection for permanent hypoparathyroidism was 5.2 times higher (*p* = 0.007), while the RR for transient hypoparathyroidism was 3.5 times higher (*p* = 0.036) (^11^).

Similarly, Giordano and cols. (^13^), in a retrospective analysis of 1,087 cases, identified permanent hypocalcemia rates of 6.3% in patients undergoing total thyroidectomy (Group A), 7% in those with thyroidectomy and elective unilateral central compartment dissection (Group B), and 16.2% in those with total thyroidectomy and bilateral central compartment dissection (Group C). There was no statistically significant difference between Groups A and B (*p* = 0.818), but a significant difference was observed between Groups A and C (*p* <0.001; OR: 2.860; 95% CI: 1.725-4.743), showing that surgical manipulation significantly affects parathyroid gland injury (^13^).

The occurrence of severe acute hypocalcemia – in- dicated by the need for continuous calcium gluconate infusion – likely signifies extensive damage to the para- thyroid glands or an additional underlying condition that impairs calcium absorption. In this study, all pa- tients who developed severe acute hypocalcemia transitioned to permanent hypoparathyroidism. Therefore, a multidisciplinary approach, including collaboration with endocrinologists, is essential to reduce long-term morbidity and improve symptom management (^14^).

In the study by Deering and cols. (^19^), analyzing 1,406 patients with definitive hypoparathyroidism and 773 with transient hypoparathyroidism, a higher prevalence of cervical dissection was observed in the definitive group (23.6% *vs.* 5.3%). During the first two years of follow-up, this group also had a higher incidence of hospitalizations (17.4% *vs.* 14.4%) and emergency room visits (26.0% *vs.* 21.4%). Among those hospitalized, they also consulted more frequently with other specialists, including endocrinologists (28.7% *vs.* 15.8%), cardiologists (16.7% *vs.* 9.7%), and nephrologists (4.6% *vs.* 3.3%) (^19^). This reinforces the need for a multidisciplinary approach in these patients. Therefore, when clear evidence shows progression to definitive hypoparathyroidism, a multidisciplinary approach should ideally be considered to reduce the need for hospitalizations due to decompensations.

This study had a retrospective cohort design, which has certain limitations, including potential loss of follow-up for some patients and the absence of data on preoperative tests, such as vitamin D levels. However, the large sample size and extended follow-up period allowed us to observe a strong correlation between the extent of cervical dissection, the severity of acute hypocalcemia, and permanent hypoparathyroidism, facilitating more personalized care for these patients through a multidisciplinary approach.

In conclusion, the incidence of permanent hypo- parathyroidism is closely associated with the extent of level VI dissection, especially in terms of the number of lymph nodes removed and whether the dissection was bilateral. Additionally, the severity of post-operative hy- pocalcemia is indicated by the decrease in PTH levels and the need for, as well as the duration of, intravenous calcium supplementation.
